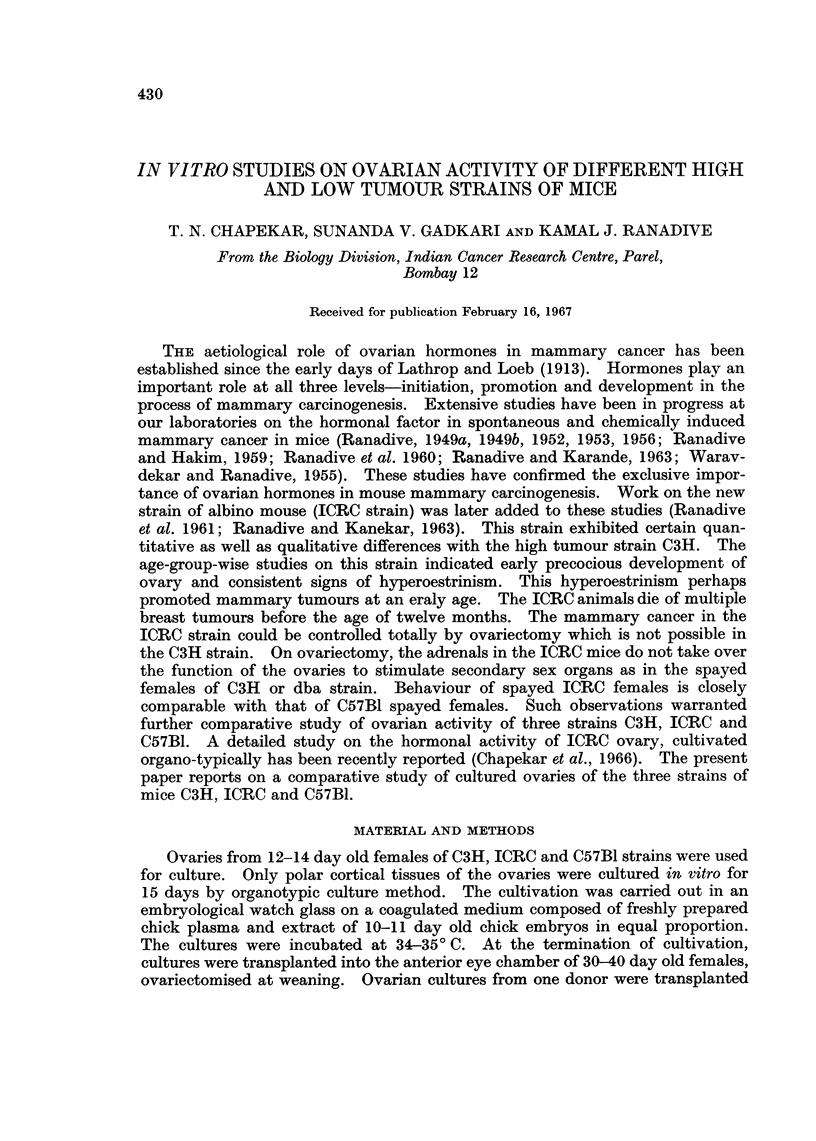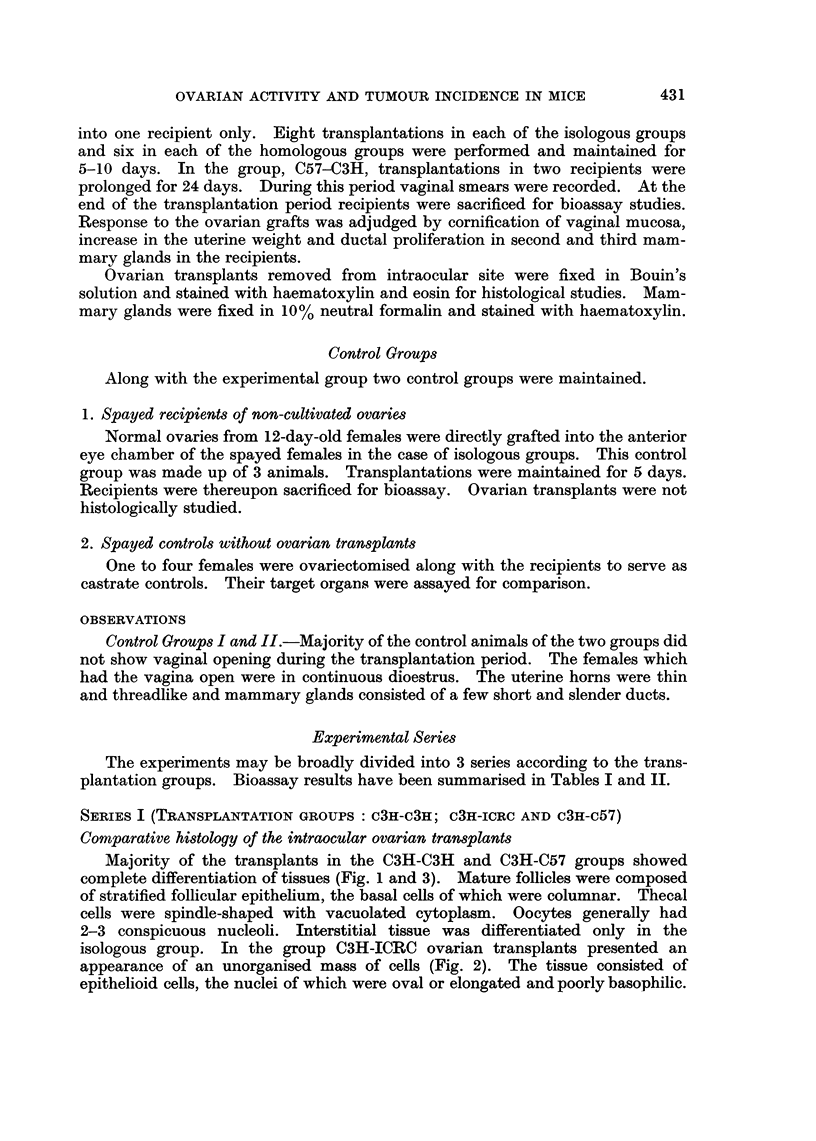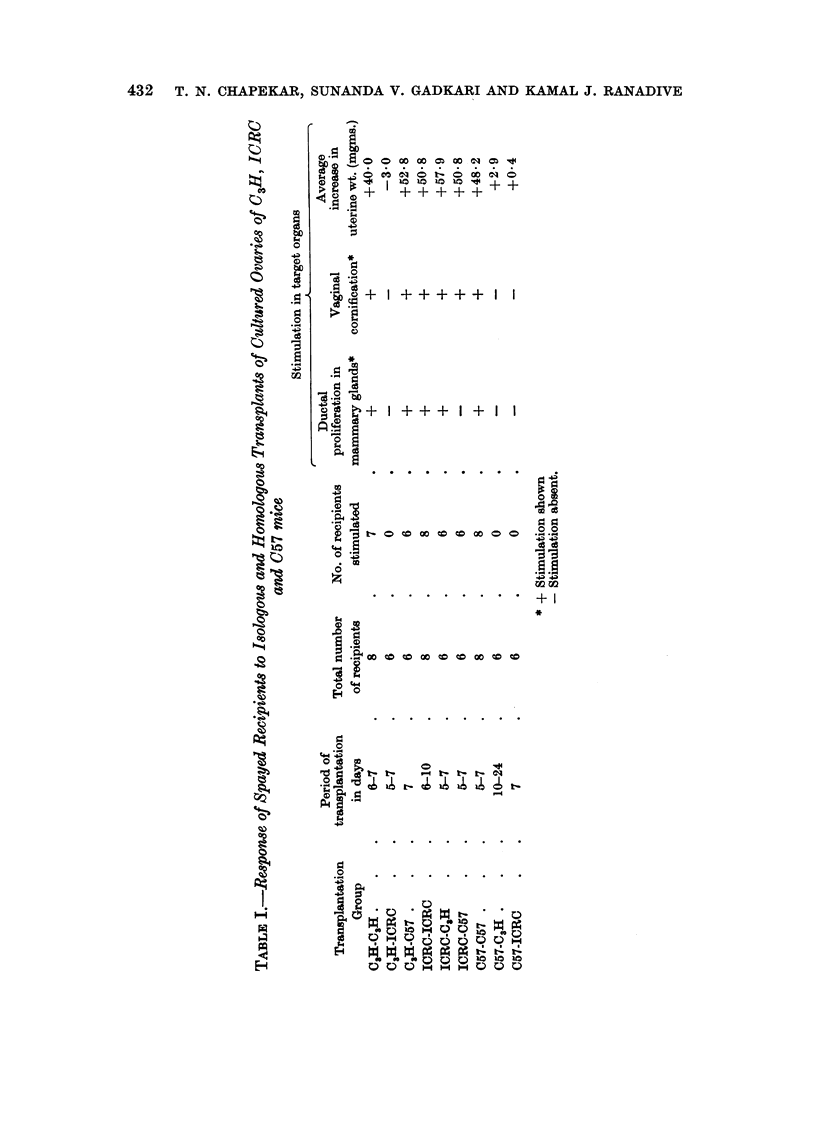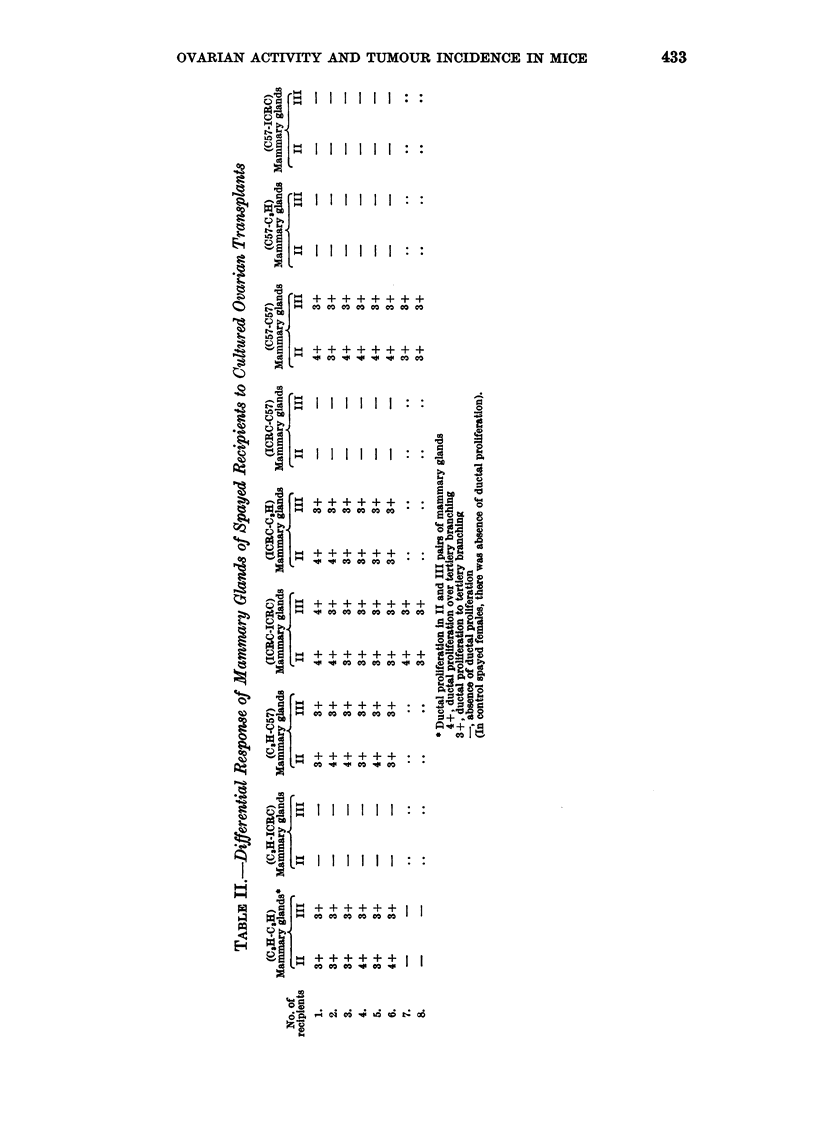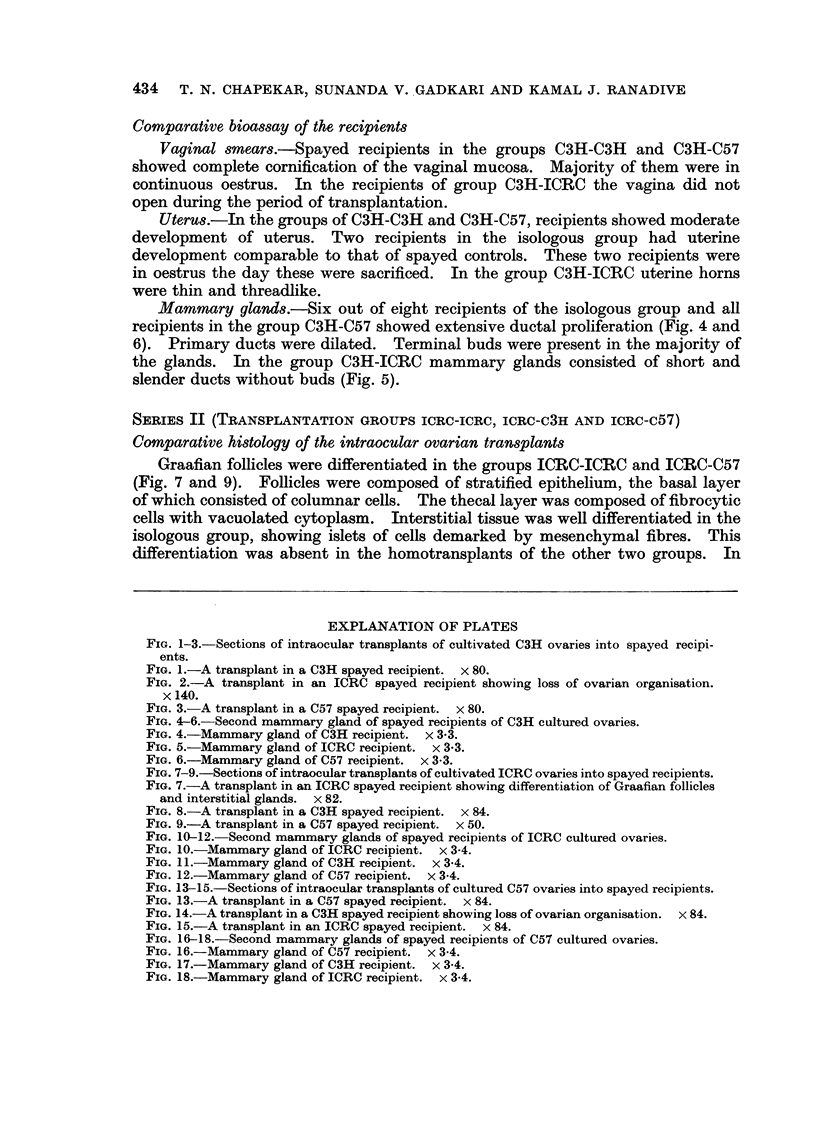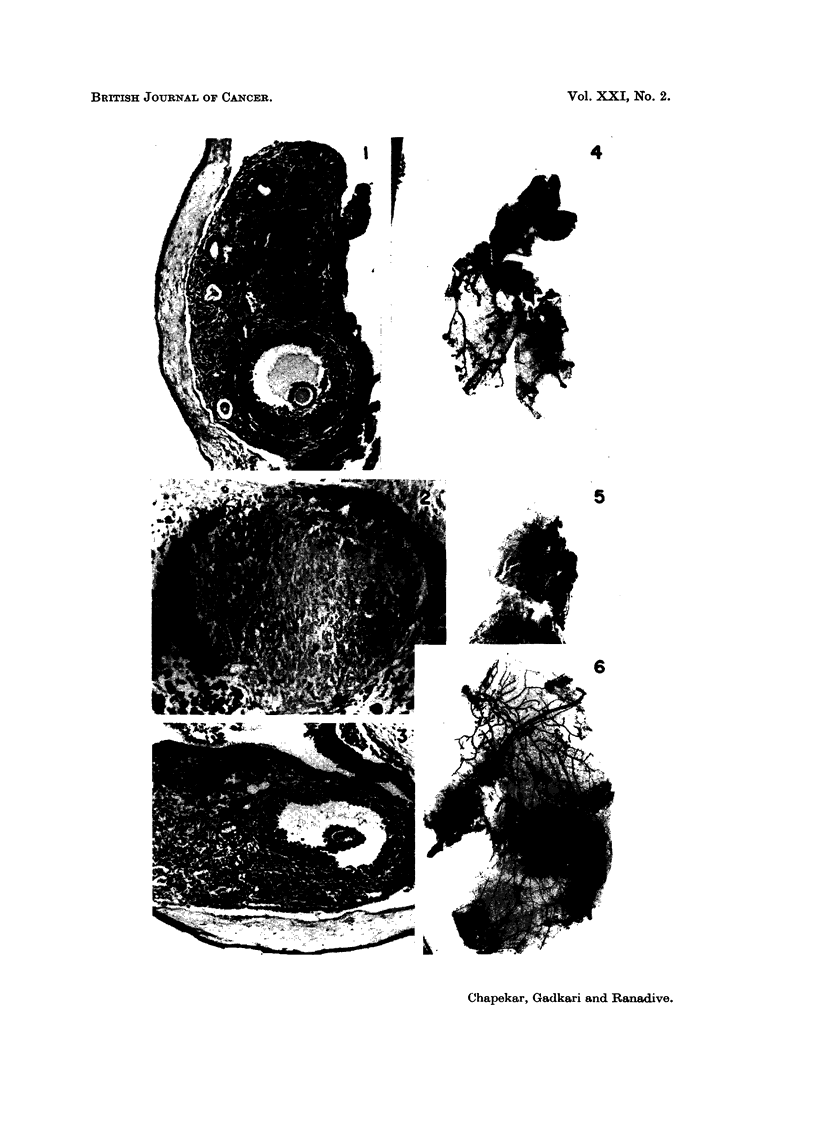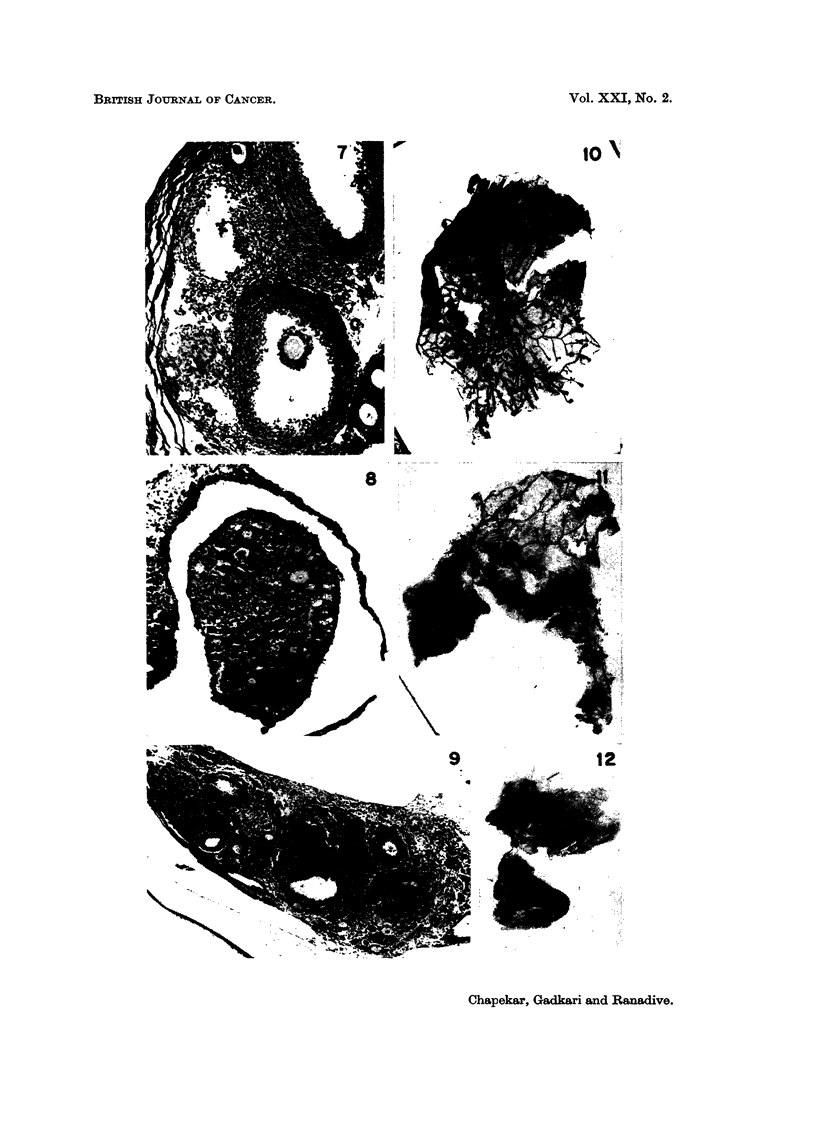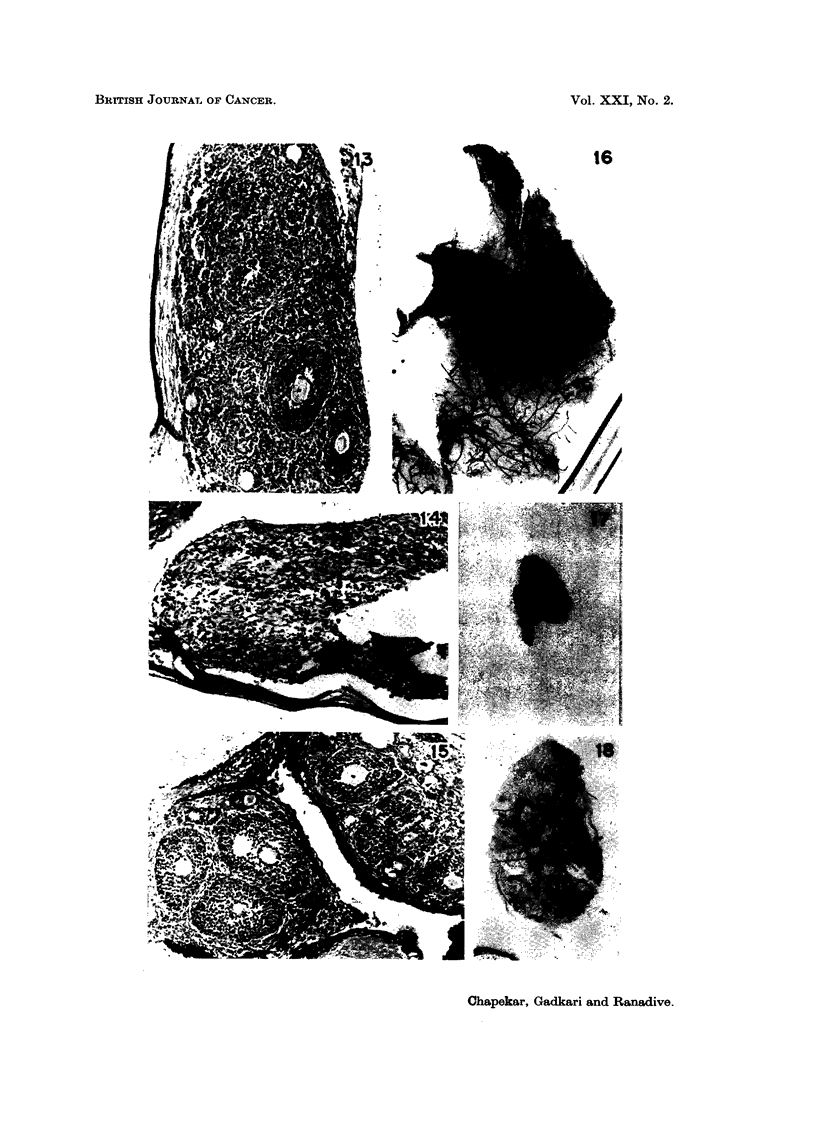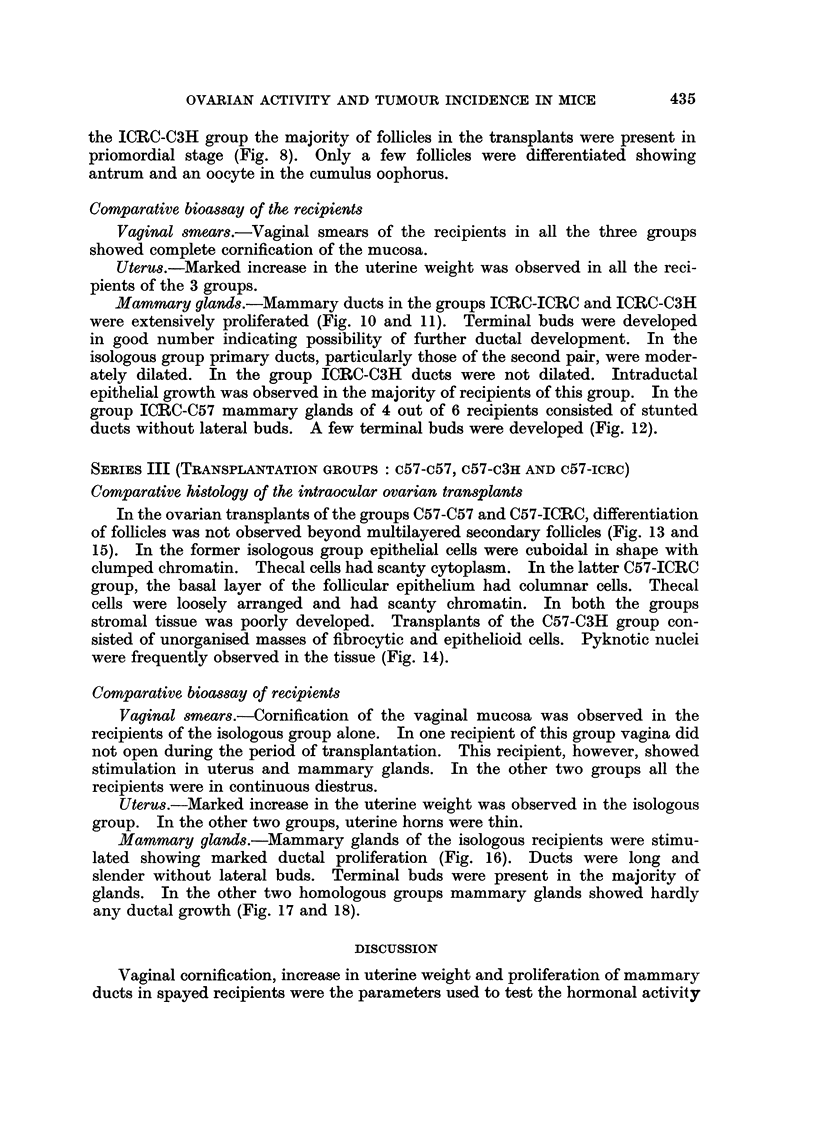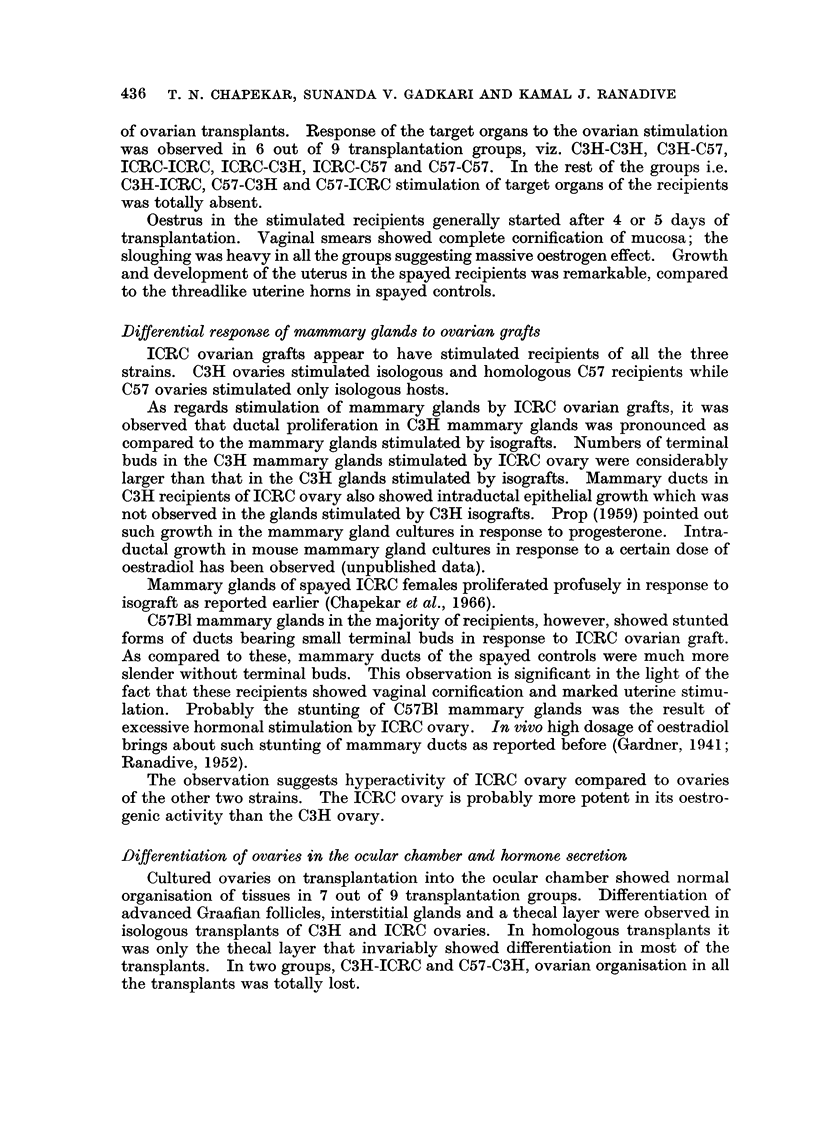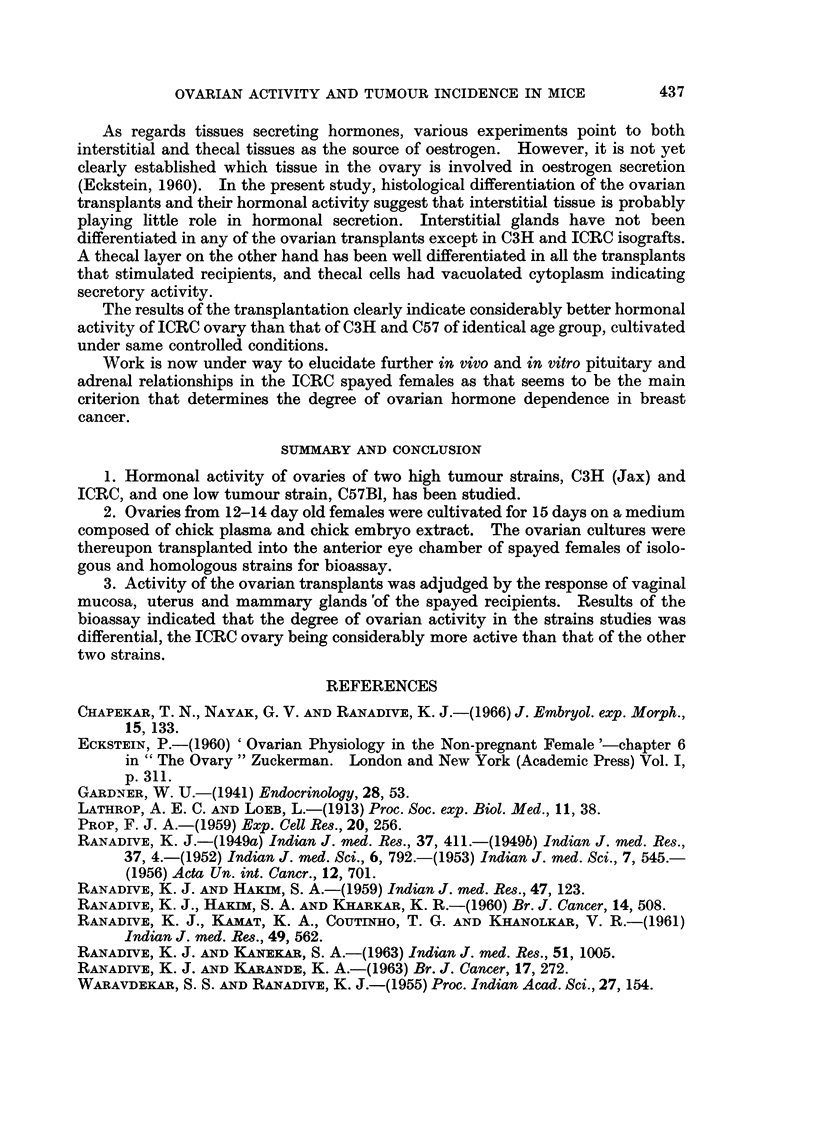# In vitro studies on ovarian activity of different high and low tumour strains of mice.

**DOI:** 10.1038/bjc.1967.49

**Published:** 1967-06

**Authors:** T. N. Chapekar, S. V. Gadkari, K. J. Ranadive

## Abstract

**Images:**


### *IN VITRO* STUDIES ON OVARIAN ACTIVITY OF DIFFERENT HIGH AND LOW TUMOUR STRAINS OF MICE

T. N. CHAPEKAR, SUNANDA V. GADKARI AND KAMAL J. RANADIVE

*From the Biology Division, Indian Cancer Research Centre, Parel, Bombay* 12

Received for publication February 16, 1967

THE aetiological role of ovarian hormones in mammary cancer has been established since the early days of Lathrop and Loeb (1913). Hormones play an important role at all three levels—initiation, promotion and development in the process of mammary carcinogenesis. Extensive studies have been in progress at our laboratories on the hormonal factor in spontaneous and chemically induced mammary cancer in mice (Ranadive, 1949*a*, 1949*b*, 1952, 1953, 1956; Ranadive and Hakim, 1959; Ranadive *et al.* 1960; Ranadive and Karande, 1963; Waravdekar and Ranadive, 1955). These studies have confirmed the exclusive importance of ovarian hormones in mouse mammary carcinogenesis. Work on the new strain of albino mouse (ICRC strain) was later added to these studies (Ranadive *et al.* 1961; Ranadive and Kanekar, 1963). This strain exhibited certain quantitative as well as qualitative differences with the high tumour strain C3H. The age-group-wise studies on this strain indicated early precocious development of ovary and consistent signs of hyperoestrinism. This hyperoestrinism perhaps promoted mammary tumours at an eraly age. The ICRC animals die of multiple breast tumours before the age of twelve months. The mammary cancer in the ICRC strain could be controlled totally by ovariectomy which is not possible in the C3H strain. On ovariectomy, the adrenals in the ICRC mice do not take over the function of the ovaries to stimulate secondary sex organs as in the spayed females of C3H or dba strain. Behaviour of spayed ICRC females is closely comparable with that of C57Bl spayed females. Such observations warranted further comparative study of ovarian activity of three strains C3H, ICRC and C57Bl. A detailed study on the hormonal activity of ICRC ovary, cultivated organo-typically has been recently reported (Chapekar *et al.*, 1966). The present paper reports on a comparative study of cultured ovaries of the three strains of mice C3H, ICRC and C57Bl.

### MATERIAL AND METHODS

Ovaries from 12–14 day old females of C3H, ICRC and C57Bl strains were used for culture. Only polar cortical tissues of the ovaries were cultured *in vitro* for 15 days by organotypic culture method. The cultivation was carried out in an embryological watch glass on a coagulated medium composed of freshly prepared chick plasma and extract of 10–11 day old chick embryos in equal proportion. The cultures were incubated at 34–35° C. At the termination of cultivation, cultures were transplanted into the anterior eye chamber of 30–40 day old females, ovariectomised at weaning. Ovarian cultures from one donor were transplanted into one recipient only. Eight transplantations in each of the isologous groups and six in each of the homologous groups were performed and maintained for 5–10 days. In the group, C57–C3H, transplantations in two recipients were prolonged for 24 days. During this period vaginal smears were recorded. At the end of the transplantation period recipients were sacrificed for bioassay studies. Response to the ovarian grafts was adjudged by cornification of vaginal mucosa, increase in the uterine weight and ductal proliferation in second and third mammary glands in the recipients.

Ovarian transplants removed from intraocular site were fixed in Bouin's solution and stained with haematoxylin and eosin for histological studies. Mammary glands were fixed in 10% neutral formalin and stained with haematoxylin.

### *Control Groups*

Along with the experimental group two control groups were maintained.

1. *Spayed recipients of non-cultivated ovaries*

Normal ovaries from 12-day-old females were directly grafted into the anterior eye chamber of the spayed females in the case of isologous groups. This control group was made up of 3 animals. Transplantations were maintained for 5 days. Recipients were thereupon sacrificed for bioassay. Ovarian transplants were not histologically studied.

2. *Spayed controls without ovarian transplants*

One to four females were ovariectomised along with the recipients to serve as castrate controls. Their target organs were assayed for comparison.

### OBSERVATIONS

*Control Groups I and II.*—Majority of the control animals of the two groups did not show vaginal opening during the transplantation period. The females which had the vagina open were in continuous dioestrus. The uterine horns were thin and threadlike and mammary glands consisted of a few short and slender ducts.

### *Experimental Series*

The experiments may be broadly divided into 3 series according to the transplantation groups. Bioassay results have been summarised in Tables I and II.

### SERIES I (TRANSPLANTATION GROUPS : C3H-C3H; C3H-ICRC AND C3H-C57)

*Comparative histology of the intraocular ovarian transplants*

Majority of the transplants in the C3H-C3H and C3H-C57 groups showed complete differentiation of tissues (Fig. 1 and 3). Mature follicles were composed of stratified follicular epithelium, the basal cells of which were columnar. Thecal cells were spindle-shaped with vacuolated cytoplasm. Oocytes generally had 2–3 conspicuous nucleoli. Interstitial tissue was differentiated only in the isologous group. In the group C3H-ICRC ovarian transplants presented an appearance of an unorganised mass of cells (Fig. 2). The tissue consisted of epithelioid cells, the nuclei of which were oval or elongated and poorly basophilic.

TABLE I.—*Response of Spayed Recipients to Isologous and Homologous Transplants of Cultured Ovaries of $C_3H$, ICRC and C57 mice*

| Transplantation Group | Period of transplantation in days | Total number of recipients | No. of recipients stimulated | Stimulation in target organs: Ductal proliferation in mammary glands* | Vaginal cornification* | Average increase in uterine wt. (mgms.) |
|---|---|---|---|---|---|---|
| $C_3H$-$C_3H$ | 6–7 | 8 | 7 | + | + | +40·0 |
| $C_3H$-ICRC | 5–7 | 6 | 0 | – | – | –3·0 |
| $C_3H$-C57 | 7 | 6 | 6 | + | + | +52·8 |
| ICRC-ICRC | 6–10 | 8 | 8 | + | + | +50·8 |
| ICRC-$C_3H$ | 5–7 | 6 | 6 | + | + | +57·9 |
| ICRC-C57 | 5–7 | 6 | 6 | – | + | +50·8 |
| C57-C57 | 5–7 | 8 | 8 | + | + | +48·2 |
| C57-$C_3H$ | 10–24 | 6 | 0 | – | – | +2·9 |
| C57-ICRC | 7 | 6 | 0 | – | – | +0·4 |

* + Stimulation shown

– Stimulation absent.

TABLE II.—*Differential Response of Mammary Glands of Spayed Recipients to Cultured Ovarian Transplants*

| No. of recipients | ($C_3$H-$C_3$H) Mammary glands* | | ($C_3$H-ICRC) Mammary glands | | ($C_3$H-C57) Mammary glands | | (ICRC-ICRC) Mammary glands | | (ICRC-$C_3$H) Mammary glands | | (ICRC-C57) Mammary glands | | (C57-C57) Mammary glands | | (C57-$C_3$H) Mammary glands | | (C57-ICRC) Mammary glands | |
|---|---|---|---|---|---|---|---|---|---|---|---|---|---|---|---|---|---|---|
| | II | III | II | III | II | III | II | III | II | III | II | III | II | III | II | III | II | III |
| 1. | 3+ | 3+ | — | — | 3+ | 3+ | 4+ | 4+ | 4+ | 3+ | — | — | 4+ | 3+ | — | — | — | — |
| 2. | 3+ | 3+ | — | — | 4+ | 3+ | 4+ | 3+ | 4+ | 3+ | — | — | 3+ | 3+ | — | — | — | — |
| 3. | 3+ | 3+ | — | — | 4+ | 3+ | 3+ | 3+ | 3+ | 3+ | — | — | 4+ | 3+ | — | — | — | — |
| 4. | 4+ | 3+ | — | — | 3+ | 3+ | 3+ | 3+ | 3+ | 3+ | — | — | 4+ | 3+ | — | — | — | — |
| 5. | 3+ | 3+ | — | — | 4+ | 3+ | 3+ | 3+ | 3+ | 3+ | — | — | 4+ | 3+ | — | — | — | — |
| 6. | 4+ | 3+ | — | — | 3+ | 3+ | 3+ | 3+ | 3+ | 3+ | — | — | 4+ | 3+ | — | — | — | — |
| 7. | — | — | .. | .. | .. | .. | 4+ | 3+ | .. | .. | .. | .. | 3+ | 3+ | .. | .. | .. | .. |
| 8. | — | — | .. | .. | .. | .. | 3+ | 3+ | .. | .. | .. | .. | 3+ | 3+ | .. | .. | .. | .. |

* Ductal proliferation in II and III pairs of mammary glands

4+, ductal proliferation over tertiery branching

3+, ductal proliferation to tertiery branching

—, absence of ductal proliferation

(In control spayed females, there was absence of ductal proliferation).

*Comparative bioassay of the recipients*

*Vaginal smears.*—Spayed recipients in the groups C3H-C3H and C3H-C57 showed complete cornification of the vaginal mucosa. Majority of them were in continuous oestrus. In the recipients of group C3H-ICRC the vagina did not open during the period of transplantation.

*Uterus.*—In the groups of C3H-C3H and C3H-C57, recipients showed moderate development of uterus. Two recipients in the isologous group had uterine development comparable to that of spayed controls. These two recipients were in oestrus the day these were sacrificed. In the group C3H-ICRC uterine horns were thin and threadlike.

*Mammary glands.*—Six out of eight recipients of the isologous group and all recipients in the group C3H-C57 showed extensive ductal proliferation (Fig. 4 and 6). Primary ducts were dilated. Terminal buds were present in the majority of the glands. In the group C3H-ICRC mammary glands consisted of short and slender ducts without buds (Fig. 5).

SERIES II (TRANSPLANTATION GROUPS ICRC-ICRC, ICRC-C3H AND ICRC-C57)

*Comparative histology of the intraocular ovarian transplants*

Graafian follicles were differentiated in the groups ICRC-ICRC and ICRC-C57 (Fig. 7 and 9). Follicles were composed of stratified epithelium, the basal layer of which consisted of columnar cells. The thecal layer was composed of fibrocytic cells with vacuolated cytoplasm. Interstitial tissue was well differentiated in the isologous group, showing islets of cells demarked by mesenchymal fibres. This differentiation was absent in the homotransplants of the other two groups. In

---

EXPLANATION OF PLATES

FIG. 1–3.—Sections of intraocular transplants of cultivated C3H ovaries into spayed recipients.
FIG. 1.—A transplant in a C3H spayed recipient. × 80.
FIG. 2.—A transplant in an ICRC spayed recipient showing loss of ovarian organisation. × 140.
FIG. 3.—A transplant in a C57 spayed recipient. × 80.
FIG. 4–6.—Second mammary gland of spayed recipients of C3H cultured ovaries.
FIG. 4.—Mammary gland of C3H recipient. × 3·3.
FIG. 5.—Mammary gland of ICRC recipient. × 3·3.
FIG. 6.—Mammary gland of C57 recipient. × 3·3.
FIG. 7–9.—Sections of intraocular transplants of cultivated ICRC ovaries into spayed recipients.
FIG. 7.—A transplant in an ICRC spayed recipient showing differentiation of Graafian follicles and interstitial glands. × 82.
FIG. 8.—A transplant in a C3H spayed recipient. × 84.
FIG. 9.—A transplant in a C57 spayed recipient. × 50.
FIG. 10–12.—Second mammary glands of spayed recipients of ICRC cultured ovaries.
FIG. 10.—Mammary gland of ICRC recipient. × 3·4.
FIG. 11.—Mammary gland of C3H recipient. × 3·4.
FIG. 12.—Mammary gland of C57 recipient. × 3·4.
FIG. 13–15.—Sections of intraocular transplants of cultured C57 ovaries into spayed recipients.
FIG. 13.—A transplant in a C57 spayed recipient. × 84.
FIG. 14.—A transplant in a C3H spayed recipient showing loss of ovarian organisation. × 84.
FIG. 15.—A transplant in an ICRC spayed recipient. × 84.
FIG. 16–18.—Second mammary glands of spayed recipients of C57 cultured ovaries.
FIG. 16.—Mammary gland of C57 recipient. × 3·4.
FIG. 17.—Mammary gland of C3H recipient. × 3·4.
FIG. 18.—Mammary gland of ICRC recipient. × 3·4.

Chapekar, Gadkari and Ranadive.

Chapekar, Gadkari and Ranadive.

**Chapekar, Gadkari and Ranadive.**

the ICRC-C3H group the majority of follicles in the transplants were present in priomordial stage (Fig. 8). Only a few follicles were differentiated showing antrum and an oocyte in the cumulus oophorus.

*Comparative bioassay of the recipients*

*Vaginal smears.*—Vaginal smears of the recipients in all the three groups showed complete cornification of the mucosa.

*Uterus.*—Marked increase in the uterine weight was observed in all the recipients of the 3 groups.

*Mammary glands.*—Mammary ducts in the groups ICRC-ICRC and ICRC-C3H were extensively proliferated (Fig. 10 and 11). Terminal buds were developed in good number indicating possibility of further ductal development. In the isologous group primary ducts, particularly those of the second pair, were moderately dilated. In the group ICRC-C3H ducts were not dilated. Intraductal epithelial growth was observed in the majority of recipients of this group. In the group ICRC-C57 mammary glands of 4 out of 6 recipients consisted of stunted ducts without lateral buds. A few terminal buds were developed (Fig. 12).

SERIES III (TRANSPLANTATION GROUPS : C57-C57, C57-C3H AND C57-ICRC)

*Comparative histology of the intraocular ovarian transplants*

In the ovarian transplants of the groups C57-C57 and C57-ICRC, differentiation of follicles was not observed beyond multilayered secondary follicles (Fig. 13 and 15). In the former isologous group epithelial cells were cuboidal in shape with clumped chromatin. Thecal cells had scanty cytoplasm. In the latter C57-ICRC group, the basal layer of the follicular epithelium had columnar cells. Thecal cells were loosely arranged and had scanty chromatin. In both the groups stromal tissue was poorly developed. Transplants of the C57-C3H group consisted of unorganised masses of fibrocytic and epithelioid cells. Pyknotic nuclei were frequently observed in the tissue (Fig. 14).

*Comparative bioassay of recipients*

*Vaginal smears.*—Cornification of the vaginal mucosa was observed in the recipients of the isologous group alone. In one recipient of this group vagina did not open during the period of transplantation. This recipient, however, showed stimulation in uterus and mammary glands. In the other two groups all the recipients were in continuous diestrus.

*Uterus.*—Marked increase in the uterine weight was observed in the isologous group. In the other two groups, uterine horns were thin.

*Mammary glands.*—Mammary glands of the isologous recipients were stimulated showing marked ductal proliferation (Fig. 16). Ducts were long and slender without lateral buds. Terminal buds were present in the majority of glands. In the other two homologous groups mammary glands showed hardly any ductal growth (Fig. 17 and 18).

### DISCUSSION

Vaginal cornification, increase in uterine weight and proliferation of mammary ducts in spayed recipients were the parameters used to test the hormonal activity of ovarian transplants. Response of the target organs to the ovarian stimulation was observed in 6 out of 9 transplantation groups, viz. C3H-C3H, C3H-C57, ICRC-ICRC, ICRC-C3H, ICRC-C57 and C57-C57. In the rest of the groups i.e. C3H-ICRC, C57-C3H and C57-ICRC stimulation of target organs of the recipients was totally absent.

Oestrus in the stimulated recipients generally started after 4 or 5 days of transplantation. Vaginal smears showed complete cornification of mucosa; the sloughing was heavy in all the groups suggesting massive oestrogen effect. Growth and development of the uterus in the spayed recipients was remarkable, compared to the threadlike uterine horns in spayed controls.

*Differential response of mammary glands to ovarian grafts*

ICRC ovarian grafts appear to have stimulated recipients of all the three strains. C3H ovaries stimulated isologous and homologous C57 recipients while C57 ovaries stimulated only isologous hosts.

As regards stimulation of mammary glands by ICRC ovarian grafts, it was observed that ductal proliferation in C3H mammary glands was pronounced as compared to the mammary glands stimulated by isografts. Numbers of terminal buds in the C3H mammary glands stimulated by ICRC ovary were considerably larger than that in the C3H glands stimulated by isografts. Mammary ducts in C3H recipients of ICRC ovary also showed intraductal epithelial growth which was not observed in the glands stimulated by C3H isografts. Prop (1959) pointed out such growth in the mammary gland cultures in response to progesterone. Intraductal growth in mouse mammary gland cultures in response to a certain dose of oestradiol has been observed (unpublished data).

Mammary glands of spayed ICRC females proliferated profusely in response to isograft as reported earlier (Chapekar *et al.*, 1966).

C57Bl mammary glands in the majority of recipients, however, showed stunted forms of ducts bearing small terminal buds in response to ICRC ovarian graft. As compared to these, mammary ducts of the spayed controls were much more slender without terminal buds. This observation is significant in the light of the fact that these recipients showed vaginal cornification and marked uterine stimulation. Probably the stunting of C57Bl mammary glands was the result of excessive hormonal stimulation by ICRC ovary. *In vivo* high dosage of oestradiol brings about such stunting of mammary ducts as reported before (Gardner, 1941; Ranadive, 1952).

The observation suggests hyperactivity of ICRC ovary compared to ovaries of the other two strains. The ICRC ovary is probably more potent in its oestrogenic activity than the C3H ovary.

*Differentiation of ovaries in the ocular chamber and hormone secretion*

Cultured ovaries on transplantation into the ocular chamber showed normal organisation of tissues in 7 out of 9 transplantation groups. Differentiation of advanced Graafian follicles, interstitial glands and a thecal layer were observed in isologous transplants of C3H and ICRC ovaries. In homologous transplants it was only the thecal layer that invariably showed differentiation in most of the transplants. In two groups, C3H-ICRC and C57-C3H, ovarian organisation in all the transplants was totally lost.

As regards tissues secreting hormones, various experiments point to both interstitial and thecal tissues as the source of oestrogen. However, it is not yet clearly established which tissue in the ovary is involved in oestrogen secretion (Eckstein, 1960). In the present study, histological differentiation of the ovarian transplants and their hormonal activity suggest that interstitial tissue is probably playing little role in hormonal secretion. Interstitial glands have not been differentiated in any of the ovarian transplants except in C3H and ICRC isografts. A thecal layer on the other hand has been well differentiated in all the transplants that stimulated recipients, and thecal cells had vacuolated cytoplasm indicating secretory activity.

The results of the transplantation clearly indicate considerably better hormonal activity of ICRC ovary than that of C3H and C57 of identical age group, cultivated under same controlled conditions.

Work is now under way to elucidate further *in vivo* and *in vitro* pituitary and adrenal relationships in the ICRC spayed females as that seems to be the main criterion that determines the degree of ovarian hormone dependence in breast cancer.

### SUMMARY AND CONCLUSION

1. Hormonal activity of ovaries of two high tumour strains, C3H (Jax) and ICRC, and one low tumour strain, C57Bl, has been studied.

2. Ovaries from 12–14 day old females were cultivated for 15 days on a medium composed of chick plasma and chick embryo extract. The ovarian cultures were thereupon transplanted into the anterior eye chamber of spayed females of isologous and homologous strains for bioassay.

3. Activity of the ovarian transplants was adjudged by the response of vaginal mucosa, uterus and mammary glands 'of the spayed recipients. Results of the bioassay indicated that the degree of ovarian activity in the strains studies was differential, the ICRC ovary being considerably more active than that of the other two strains.

### REFERENCES

Chapekar, T. N., Nayak, G. V. and Ranadive, K. J.—(1966) *J. Embryol. exp. Morph.*, **15**, 133.

Eckstein, P.—(1960) ' Ovarian Physiology in the Non-pregnant Female '—chapter 6 in " The Ovary " Zuckerman. London and New York (Academic Press) Vol. I, p. 311.

Gardner, W. U.—(1941) *Endocrinology*, **28**, 53.

Lathrop, A. E. C. and Loeb, L.—(1913) *Proc. Soc. exp. Biol. Med.*, **11**, 38.

Prop, F. J. A.—(1959) *Exp. Cell Res.*, **20**, 256.

Ranadive, K. J.—(1949*a*) *Indian J. med. Res.*, **37**, 411.—(1949*b*) *Indian J. med. Res.*, **37**, 4.—(1952) *Indian J. med. Sci.*, **6**, 792.—(1953) *Indian J. med. Sci.*, **7**, 545.—(1956) *Acta Un. int. Cancr.*, **12**, 701.

Ranadive, K. J. and Hakim, S. A.—(1959) *Indian J. med. Res.*, **47**, 123.

Ranadive, K. J., Hakim, S. A. and Kharkar, K. R.—(1960) *Br. J. Cancer*, **14**, 508.

Ranadive, K. J., Kamat, K. A., Coutinho, T. G. and Khanolkar, V. R.—(1961) *Indian J. med. Res.*, **49**, 562.

Ranadive, K. J. and Kanekar, S. A.—(1963) *Indian J. med. Res.*, **51**, 1005.

Ranadive, K. J. and Karande, K. A.—(1963) *Br. J. Cancer*, **17**, 272.

Waravdekar, S. S. and Ranadive, K. J.—(1955) *Proc. Indian Acad. Sci.*, **27**, 154.